# Ultra-low-dose CT reconstructed with the artificial intelligence iterative reconstruction algorithm (AIIR) in ^18^F-FDG total-body PET/CT examination: a preliminary study

**DOI:** 10.1186/s40658-022-00521-8

**Published:** 2023-01-02

**Authors:** Yan Hu, Zhe Zheng, Haojun Yu, Jingyi Wang, Xinlan Yang, Hongcheng Shi

**Affiliations:** 1 Department of Nuclear Medicine, Zhongshan Hospital, Fudan University, 180 Fenglin Rd, Shanghai, 200032 China; 2 Nuclear Medicine Institute of Fudan University, Shanghai, 200032 China; 3 Shanghai Institute of Medical Imaging, Shanghai, 200032 China; 4 United Imaging Healthcare Co., Ltd., Shanghai, China

**Keywords:** Ultra-low-dose, Standard-dose, Artificial intelligence iterative reconstruction, PET/CT

## Abstract

**Purpose:**

To investigate the feasibility of ultra-low-dose CT (ULDCT) reconstructed with the artificial intelligence iterative reconstruction (AIIR) algorithm in total-body PET/CT imaging.

**Methods:**

The study included both the phantom and clinical parts. An anthropomorphic phantom underwent CT imaging with ULDCT (10mAs) and standard-dose CT (SDCT) (120mAs), respectively. ULDCT was reconstructed with AIIR and hybrid iterative reconstruction (HIR) (expressed as ULDCT-AIIR_phantom_ and ULDCT-HIR_phantom_), respectively, and SDCT was reconstructed with HIR (SDCT-HIR_phantom_) as control. In the clinical part, 52 patients with malignant tumors underwent the total-body PET/CT scan. ULDCT with AIIR (ULDCT-AIIR) and HIR (ULDCT-HIR), respectively, was reconstructed for PET attenuation correction, followed by the SDCT reconstructed with HIR (SDCT-HIR) for anatomical location. PET/CT images’ quality was qualitatively assessed by two readers. The CT_mean_, as well as the CT standard deviation (CT_sd_), SUV_max_, SUV_mean_, and the SUV standard deviation (SUV_sd_), was recorded. The signal-to-noise ratio (SNR) and contrast-to-noise ratio (CNR) were calculated and compared.

**Results:**

The image quality of ULDCT-HIR_phantom_ was inferior to the SDCT-HIR_phantom_, but no significant difference was found between the ULDCT-AIIR_phantom_ and SDCT-HIR_phantom_. The subjective score of ULDCT-AIIR in the neck, chest and lower limb was equivalent to that of SDCT-HIR. Besides the brain and lower limb, the change rates of CT_mean_ in thyroid, neck muscle, lung, mediastinum, back muscle, liver, lumbar muscle, first lumbar spine and sigmoid colon were −2.15, −1.52, 0.66, 2.97, 0.23, 8.91, 0.06, −4.29 and 8.78%, respectively, while all CT_sd_ of ULDCT-AIIR was lower than that of SDCT-HIR. Except for the brain, the CNR of ULDCT-AIIR was the same as the SDCT-HIR, but the SNR was higher. The change rates of SUV_max_, SUV_mean_ and SUV_sd_ were within $\pm$ 3% in all ROIs. For the lesions, the SUV_max_, SUV_sd_ and TBR showed no significant difference between PET-AIIR and PET-HIR.

**Conclusion:**

The SDCT-HIR could not be replaced by the ULDCT-AIIR at date, but the AIIR algorithm decreased the image noise and increased the SNR, which can be implemented under special circumstances in PET/CT examination.

**Supplementary Information:**

The online version contains supplementary material available at 10.1186/s40658-022-00521-8.

## Background

PET/CT noninvasively provides both physiological and anatomical information in vivo in a single gantry, which has become a powerful imaging modality in the diagnosis, staging, prognosis, treatment planning, and therapy response assessment [1–3]. Recently, concerns about radiation-exposure-induced carcinogenesis in PET/CT examination have been raised, especially for patients undergoing repeat scans for treatment evaluation. The trade-off between the diagnostic need and the radiation exposure in the PET/CT examination should be balanced, particularly for pediatric patients [4, 5]. With the advance of the total-body PET/CT scanners with the improved sensitivity, studies have proven to have the potential of fast and ultra-low-dose PET imaging in the clinical practice [6–8].

The CT protocols used in PET/CT can be divided into three types according to the different applications: diagnosis, anatomical localization and only for PET attenuation correction. CT dose was the major contributor to the effective activity in PET/CT procedures [9]. CT dose reduction techniques such as tube current modulation, tube voltage reduction, pitch increment and noise reduction filters, and new reconstruction algorithms were proposed and implemented in the past years [10] [11]. Among them, adaptive statistical iterative reconstruction algorithm, such as HIR, were implemented for dose reduction and serves as a regular reconstruction algorithm in the daily clinical. It consists of a scanner model, a statistical noise model, and projection noise estimation, which optimizes raw data in the projection domains through iteration loops [12]. The HIR suppresses the image noise, but may lead to “plastic” images and degrade diagnostic accuracy [13]. The AIIR, a deep-learning-based reconstruction algorithm, was originally trained with standard and stimulated multi-dose level CT image aiming to generate images with lower noise and to improve low contrast detectability [14]. This newly developed reconstruction algorithm is capable of denoising low-dose images while maintaining the image quality compared to the SDCT images [14–16]. Thus, the radiation exposure could be theoretically further reduced with the AIIR, but few studies on ULDCT scans have been conducted.

This study aims to investigate the potential of AIIR in dose reduction in the total-body PET/CT system retrospectively, and compare the image quality between the AIIR-reconstructed ULDCT and HIR-reconstructed SDCT images.

## Material and methods

### Phantom study

#### Phantom acquisition and reconstruction

An anthropomorphic phantom, Kyoto Kagaku PBU-60 (Kyoto Kagaku Co., Kyoto, Japan), was assembled according to the instruction manual, and placed in the head-first position. The phantom was scanned in two dose levels, which were consistence with the clinical acquisition protocols for ULDCT and SDCT. The CT images were reconstructed with AIIR and HIR with the convolution kernel (level 5), B-Soft-B (United Imaging Healthcare, China) for ULDCT: ULDCT-HIR_phantom_ and ULDCT-AIIR_phantom_, and SDCT-HIR_phantom_ as control.

#### Phantom data analysis

Ten ROIs were drawn on CT images at the white matter, gray matter, CSF, thoracic vertebra, heart, air in the lung, liver, kidney, muscle, and femur of the anthropomorphic phantom. The image signal (CT_mean_) and image noise (CT_sd_) of the ROIs were statistically assessed. The SNR and CNR were calculated for each ROI to compare the image quality for each group defined as follows: $SNR= \frac{{CT}_{mean, ROI}}{{CTsd}_{, ROI}}$ [17] $CNR= \frac{{CT}_{mean,ROI}-{CT}_{mean, Ref}}{({CT}_{sd,ROI}+{CT}_{sd,Ref})/2}$ [18] with muscle as reference.

### Clinical study

#### Study population

This retrospective study was approved by the Institutional Review Board of Zhongshan Hospital, Fudan University, and written informed consent was signed for the use of the data from all the subjects. 52 patients (mean age 59.48 ± 13.62; 29 male, range 41–86 years; 23 female, range 21–86 years) were randomly enrolled in this study from October to November 2021 (Table 1). The exclusion criteria included: no uptake of FDG in the primary lesions, blood glucose level > 11.0 mmol/L, an FDG uptake time of more than 70 min, and the images severely affected by the motion artifact.

**Table 1:** Demographics of patients who underwent total-body PET/CT with AIIR reconstructions

Characteristic	Value
Age (year, range)	59.48 ± 13.62 (21–86)
*Sex*	
Male (%)	29 (55.8%)
Female (%)	23 (44.2%)
BMI	23.37 ± 2.74 (18.01–30.26)
Blood glucose (mmol/L)	5.90 ± 1.22 (3.4–10.4)
Injected activity (MBq/kg)	120.17 ± 16.53 (76.22–157.99)
*Location of primary tumors*	
Head and neck	2 (3.85%)
Chest	16 (30.77%)
Abdomen and pelvic	33 (63.46%)
Lower limb	1 (1.92%)

#### Image acquisition

The patients fasted for at least 6 h before ^18^F-FDG administration and rested for approximately 60 min in a quiet environment after the injection. The examinations were performed on the total-body PET/CT scanner, uEXPLORER (United Imaging Healthcare), equipped with a 128-slice CT. The patient lay in the supine position and underwent CT scout for the correction of the positioning, then followed by an ACCT for the PET attenuation correction which was used as ULDCT in this study. The PET images were acquired in 10 min. After the acquisition of the PET data, the range of SDCT for the anatomical location was scanned from the top of the skull to the middle femur. The scanning protocol of the total-body ULDCT was set at the tube voltage of 120 kV, tube current of 10 mAs, gantry rotation time of 0.5 s, beam pitch of 1.0125, slice thickness of 1.0 mm and field of view of 500 mm. The acquisition protocol of SDCT was tube voltage 120 kV, auto modulation tube current with reference 120 mAs, gantry rotation time 0.5 s, beam pitch 0.9875, slice thickness 1.0 mm, field of view 500 mm.

#### Image reconstruction

The ULDCT images were reconstructed with AIIR and HIR using the convolution kernel (B-Soft-B, United Imaging Healthcare), expressed as: ULDCT-AIIR and ULDCT-HIR. The SDCT images were reconstructed by HIR with convolution kernel and served as reference, noted as SDCT-HIR. PET reconstructions were performed using the OSEM algorithm with the following parameters: TOF and PSF modeling, 3 iterations, and 20 subsets, matrix of 192 × 192, slice thickness of 1.443 mm, and a full width at half maximum of the Gaussian filter function of 3 mm. The attenuation of PET images was corrected by the ULDCT-AIIR and ULDCT-HIR, respectively (expressed as PET-AIIR and PET-HIR).

### Subjective image analysis

Two nuclear medicine physicians with 5 and 7 years of experience were blinded to the dose and reconstruction methods and viewed the images using standard window settings. The images were graded on the scale of 1–5 in the aspect of the image noise, conspicuity of anatomical structures, and diagnostic confidence for all the images from each subgroup. The conspicuity of anatomical structures includes the visualization of the brain, neck, chest, abdomen, pelvic and lower limb. The subjective score was evaluated according to [19] 1: non-diagnostic, unacceptable quality, impossible to detect important anatomic structures (clearly evaluate their margin or internal characteristics) due to high noise or severe artifacts; 2: poor image quality, possible to detect crucial anatomic structures (difficult to clearly evaluate their margin or internal characteristics) with distinctly increased noise or considerable artifacts; 3: acceptable image quality, depiction of the anatomic structures (margin or internal characteristics can be detected) with noticeable noise or some distinct artifacts, may obscure very subtle details; 4: good image quality, depiction of all anatomic structures (easier to evaluate their margin or internal characteristics) with slightly increased noise, above average image quality with the noise and artifacts not affecting diagnostic value;and 5: excellent image quality, depiction of all anatomic structures with fine details (significantly easier to evaluate their margin or internal characteristics, without any indistinct findings), free of artifacts and with imperceptible noise.

### Objective image analysis

For all three groups, ULDCT-HIR, ULDCT-AIIR, and SDCT-HIR in the clinical study, three ROIs were drawn at the brain, i.e., white matter, gray matter, CSF; two ROIs at the neck, i.e., thyroid, neck muscles; three ROIs were drawn on the chest, i.e., lung, mediastinum, back muscle; five were drawn at the abdomen, i.e., liver, lumbar muscle, first lumbar spine, sigmoid colon, gluteus maximus; two were in the lower limb, i.e., muscles, lower limb bone; the primary lesion was also delineated from each patient. Objective image quality for the CT images was also assessed with the CT_mean_, CT_sd_, SNR and CNR. The muscles were considered as reference in each section of the body for the calculation of CNR. The SUV_max_, SUV_mean_ and its standard deviation (SUV_sd_) were measured for PET images. TBR was calculated as follows: $TBR= \frac{{SUV}_{max, lesion}}{{SUV}_{mean, liver}}$.

### Statistical analysis

Statistical analysis was conducted using R software version 4.1.2 (R Core Team) and GraphPad Prism 6.0 (GraphPad Software Inc.). The results of objective assessment were expressed as mean $\pm$ SD. The significance of difference in the objective evaluation between each subgroup was measured by the Wilcoxon signed rank with Bonferroni correction to minimize the possibility of significance brought by multiple statistical testing. A *p* value < 0.05 was considered statistically significant. The inter-observer agreement was analyzed by the Cohen’s kappa test where the *k* value in the range of 0.00–0.20 indicated poor agreement; *k* value in the range of 0.21–0.40 indicated fair agreement; *k* value in the range of 0.41–0.60 indicated moderate agreement; *k* value in the range of 0.61–0.80 indicated good agreement, k value in the range of 0.81–1.00 indicated excellent agreement. Pearson correlation coefficient (r) was used to measure the linear relationship between the SUV_max_, SUV_mean_ and SUV_sd_ of PET-HIR and PET-AIIR. The r between 0.8 and 1.0 indicated a very strong linear relationship.

## Results

### Phantom study

**Fig. 1:**
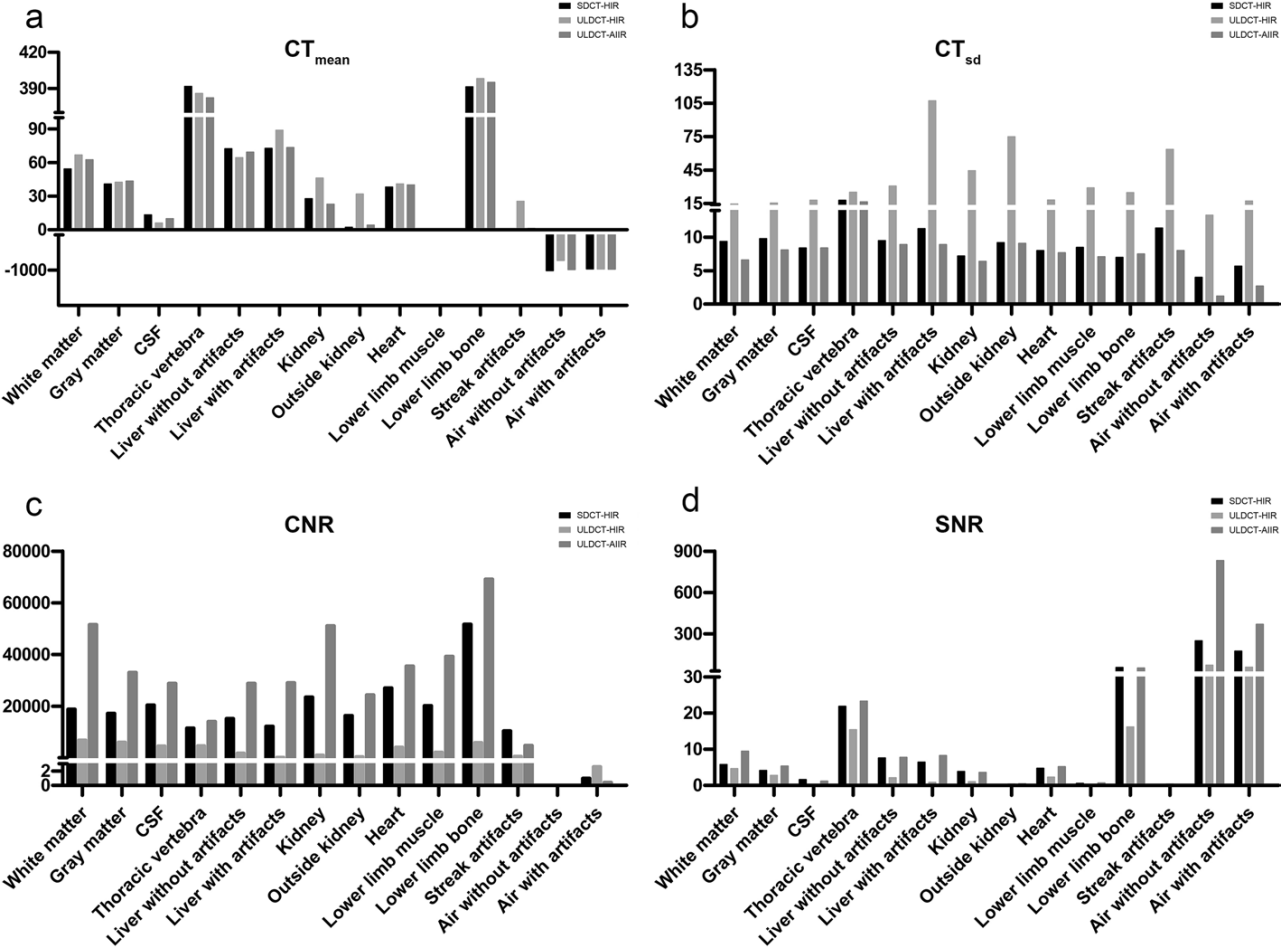
Box plot of objective measurements on phantom among the SDCT-HIR, ULDCT-HIR and ULDCT-AIIR. (**a**–**d**): The CT_mean_, CTsd, CNR and SNR of the SDCT-HIR, ULDCT-HIR and ULDCT-AIIR

**Fig. 2:**
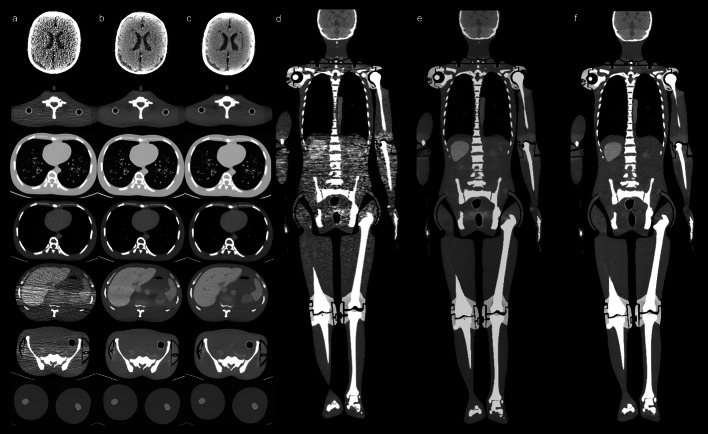
CT images of the anthropomorphic phantom with ULDCT and SDCT scanning. (**a**–**c**) column: the transverse section of different anatomy location of ULDCT-HIRphantom, ULDCT-AIIRphantom and SDCT-HIRphantom. (**d**–**f**): the coronal section of ULDCT-HIRphantom, ULDCT-AIIRphantom and SDCT-HIRphantom

The CT_mean_ of ULDCT-AIIR_phantom_ was equivalent to that of SDCT-HIR_phantom_ (*p* = 0.761), while the ULDCT-HIR_phantom_ was significantly different from that of SDCT-HIR_phantom_ (*p* = 0.049). No statistical difference was found between the image noise of SDCT-HIR_phantom_ and ULDCT-AIIR_phantom_ (*p* = 0.241), while the CT_sd_ of ULDCT-HIR_phantom_ is significantly higher than that of SDCT-HIR_phantom_ (*p* < 0.001). There is no significant difference between SDCT-HIR_phantom_ and ULDCT-AIIR_phantom_ regarding to SNR and CNR (*p* = 0.058, *p* = 0.241). However, the SNR and CNR of ULDCT-HIR_phantom_ were inferior to the SDCT-HIR_phantom_ (both *p* < 0.001) (Fig. 1). As shown in Fig. 2, the ULDCT-HIR_phantom_ was eliminated from the comparison regarding the CT image quality in the subsequent clinical experiment due to its heavy noise.

### Clinical validation

#### Subjective image analysis

**Fig. 3:**
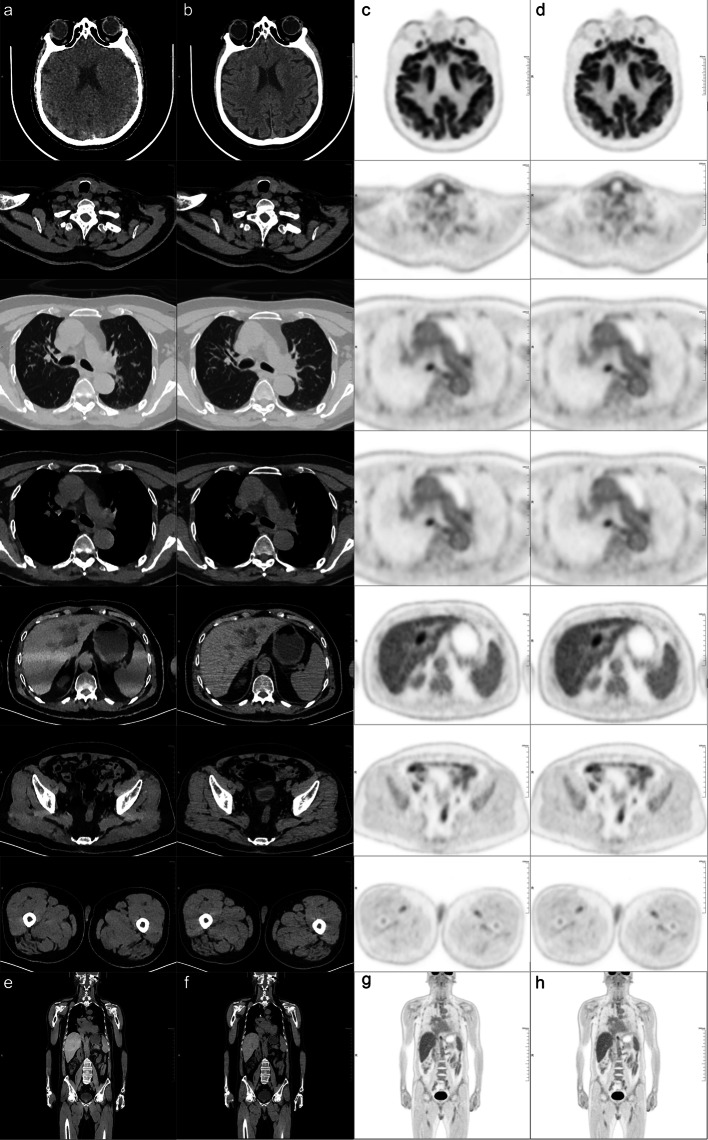
PET/CT images of a 66-year-old patient with intrahepatic cholangiocarcinoma. (**a**, **b**) column: transverse views of the CT images with ULDCT-AIIR and SDCT-HIR. **c**, **d** column: transverse views of the PET images with PET-AIIR and PET-HIR. **e**, **f**: coronal slice of the CT images with ULDCT-AIIR and SDCT-HIR. **g**, **h**: coronal slice of the PET images with ULDCT-AIIR and SDCT-HIR

The subjective scores of two CT images showed good agreement between two nuclear medicine physicians (*k* = 0.755). Compared with the SDCT-HIR, ULDCT-AIIR achieved the equivalent image quality in the neck, chest and lower limb. While the subjective scores dropped in the brain (3.81 ± 0.26 to 2.23 ± 0.33) and abdomen (3.86 ± 0.27 to 2.17 ± 0.34) in ULDCT-AIIR (Table 2). As shown in Fig. 3, the anatomical details in ULDCT-AIIR images were more obscure than that in SDCT-HIR images.

**Table 2:** Inter-rater consistency in the subjective assessment of the image qualities among the anatomical structure subgroups

Index	Subjective scores HIR	Subjective scores AIIR	Weighted Kappa
Overall	3.81 ± 0.41	3.18 ± 0.90	0.755
Brain	3.81 ± 0.26	2.23 ± 0.33	0.554
Neck	3.43 ± 0.47	3.65 ± 0.47	0.829
Chest	4.05 ± 0.42	4.03 ± 0.44	0.743
Abdomen	3.86 ± 0.27	2.17 ± 0.34	0.744
Lower limb	3.89 ± 0.29	3.80 ± 0.37	0.677

#### Objective image analysis

**Fig. 4:**
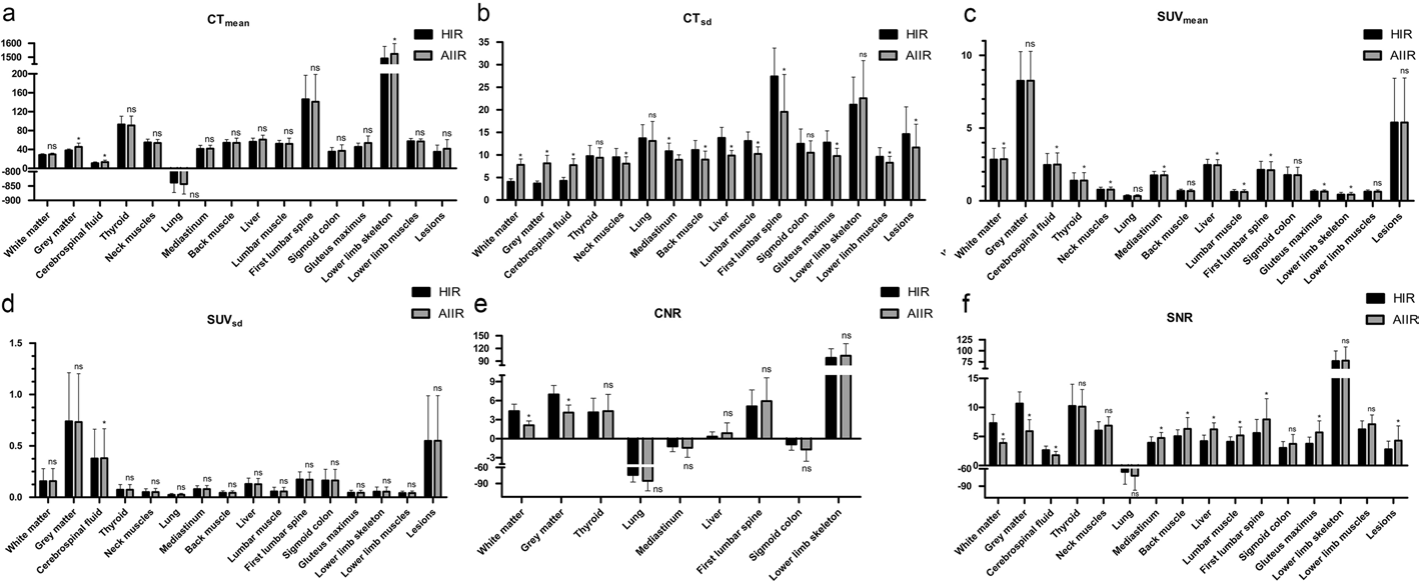
Box plot of objective parameters of ULDCT-AIIR and SDCT-HIR. (*p < 0.05; ns no significance)

As shown in Fig. 4, the CT_mean_ of the lesion, neck, chest, abdomen and pelvic in ULDCT-AIIR was equivalent to that in SDCT-HIR (all *p* > 0.05), while significant difference was found in the gray matter, cerebrospinal fluid, and lower limb skeleton (*p* < 0.05). Other than in brain, thyroid, lung, sigmoid colon, and lower limb skeleton, CT_sd_ of the ULDCT-AIIR images was lower than that of the SDCT-HIR images. The CT_sd_ of ULDCT decreased in the neck muscle (−10.80%), back muscle (−17.08%), mediastinum (−15.77%), liver (−26.74%), lumbar muscle (−20.28%,), first lumbar spine (−27.84%), gluteus maximus (−21.02%), lower limb muscles (−10.63%), and lesion (−12.09%). Besides the brain, neck, lung, sigmoid colon and lower limb, the SNR of the ULDCT-AIIR was higher than that of the SDCT-HIR (*p* < 0.001). The CNR of the ULDCT-AIIR in brain has statistically significant difference with SDCT-HIR (*p* < 0.001), but no significant difference was found in other regions.

Compared with the PET-HIR, the SUV_max_, SUV_mean_ and SUV_sd_ of PET-AIIR slightly changed in the whole body and in lesions, although significant differences were found in some anatomical locations. The change rates of SUV_max_, SUV_mean_ and SUV_sd_ of the ROIs were as follows: white matter (1.16, 1.19, 0.62%), gray matter (0.20, 0.10, −0.72%), cerebrospinal fluid (1.12, 1.23, 1.40%), thyroid (0.82, 0.95, 2.21%), neck muscle (1.33, 1.28, 0.30%), lung (0.25, 0.22, 0.96%), mediastinum (0.33, 0.42, 0.76%), back muscle (−0.38, −0.48, −0.10%), liver (−0.53, −0.52, −1.23%), lumbar muscle (−0.99, −1.43, 0.26%), first lumbar spine (−1.09, −1.14, −1.08%), sigmoid colon (−0.48, −0.51, 0.82%), gluteus maximus (−1.57, −1.51, 1.92%), lower limb skeleton (−0.63, −0.90, −0.63%), lower limb muscles (−0.01, −0.17, 0.34%). The correlation analysis showed a high degree of consistency of these parameters between the PET-HIR and PET-AIIR (*r* > 0.99, Additional file 1). For the lesions, the SUV_max_, SUV_sd_ and TBR were equivalent between PET-AIIR and PET-HIR (Table 3). Besides the cerebrospinal fluid, the SUV_sd_ between the PET-AIIR and PET-HIR showed no statistical difference.

**Table 3:** Comparisons of objective measurements on lesions between the HIR and AIIR reconstruction

Index	HIR	AIIR	Change rate (%)	*P* value
*Lesions*				
CT_mean_	35.08 ± 14.07	41.55 ± 19.33	25.87	0.523
CT_sd_	14.61 ± 6.04	11.63 ± 5.18	−12.09	0.028
SUV_max_	6.08 ± 3.50	6.06 ± 3.49	−0.27	1
SUV_mean_	5.38 ± 3.05	5.38 ± 3.07	−0.15	1
SUV_sd_	0.55 ± 0.44	0.55 ± 0.44	−0.82	1
TBR	2.53 ± 1.60	2.54 ± 1.60	0.26	1
SNR	2.79 ± 1.41	4.30 ± 2.52	71.91	0.003

## Discussion

The reduction in radiation dose from PET-CT studies is vital for pediatric patients and the patients who often undergo multiple follow-up studies [20]. It can be achieved by reducing the amount of the injected radiopharmaceutical and optimizing the CT dose in the PET/CT examination [21, 22]. Optimization of X-ray tube current and tube voltage can reduce radiation exposure, and the advanced reconstruction algorithms [23, 24] can achieve the comparable image quality with lower dose. When CT is only used for PET attenuation correction, the range of exposure settings is extremely wide [25]. Careful consideration of the acquisition parameters is required to achieve low-dose image with acceptable quality. PET/CT scanner has imaging protocols with a default value of tube current, with an option to change it to suit the patient study and the radiation doses should be kept as low as reasonably achievable. Perfect CT images, which are more appealing to our eyes, however, may not add extra diagnostic information and are obtained at the cost of giving unnecessary radiation burden to the patient [26].

The ULDCT was used as attenuation correction for total-body PET/CT in our clinic institution, which added slightly additive radiation compared to the conventional PET/CT. The ULDCT reconstructed with AIIR algorithm was evaluated in this study to explore the possibility of further dose reduction in the total-body PET/CT examination. The phantom study demonstrated that the AIIR reconstruction algorithm in ULDCT_phantom_ did not affect the CT_mean_ value, while the AIIR algorithm reduced the noise and improved the SNR of ULDCT images, and had the same level compared with SDCT-HIR_phantom_. This phantom study confirmed the feasibility of AIIR algorithm in ULDCT. In the clinical study, the image quality of ULDCT-AIIR in the neck, chest and lower limb was equivalent to the SDCT-HIR. In the brain and the abdomen, the details in ULDCT-AIIR were more obscure than those in SDCT-HIR, that is, the hands of the patient were placed on the sides of the body in this study. Thus, it was difficult for X-ray to penetrate the skull and bilateral upper limbs with high density. Obviously, the serious streaking artifact was caused by the photon starvation [27]. The attenuation was greatest and insufficient photons reached the detectors, which produced very noisy projections at the tube angulations and resulted in the horizontal artifacts. To some extent, this may affect the CT_mean_ and SNR of these parts. The streaking artifacts will be alleviated with sufficient tube current, but the patients will receive unnecessary dose of radiation in this way [28]. Noted that CT_sd_ in some anatomical parts in ULDCT with AIIR was lower than that with HIR-reconstructed SDCT images. Besides the brain, the CNR with ULDCT-AIIR was equivalent to that with SDCT-HIR. This indicated that the noise of ULDCT images was greatly decreased by the AIIR, and the CNR was kept as high as possible. The difference between the phantom and clinic study was that the phantom was a relatively ideal model, which derived from the standard patient morphology, biokinetics and organ sizes [29] and cannot completely represent the precise tissue structure of human body.

Theoretically, the tube current reduction should have a limited effect on the SUV_mean_ or SUV_max_, and the attenuation correction CT often requires less tube current to generate a low-resolution attenuation map [24]. Kumar et al. found that the SUV_mean_ varies from 1.2 to 1.4 with the changing of tube current, but the change in tube current did not change much in the SUV_sd_, and Fahey et al. found that CT noise yielded a 2% variation in SUV_sd_ [26, 30]. However, the attenuation correction CT image for PET was the ULDCT-AIIR and ULDCT-HIR, respectively, which was only related on the reconstruction algorithm. We found that the SUV_mean_ in the brain and neck was increased in PET-AIIR group but decreased in the chest and abdomen compared to that of the PET-HIR group, though these slight fluctuations had no practically clinical significance. Except for the cerebrospinal fluid, the SUV_sd_ showed no significant change between two groups, meaning that the noise of PET images does not change with the reconstruction algorithm. For the lesions, TBR between two groups also showed no significant difference, the reconstruction algorithm was not its contributory factor. This finding was similar to the previous published study, which reported that reconstruction algorithm had no change in SUV [31].

Some limitations exist in our study. Firstly, the patients were not grouped by the weight, but the dose of CT was unified. The effective activity in body could be affected by the individual differences [17, 19]. Secondly, the positioning in our study has affected the image quality and the measurement of lesion. The high-density and the low-density strip artifact was caused by the passage of high-energy X photons and the starvation of photons, respectively. Further studies may be conducted to determine the suitable positioning for total-body PET/CT. Thirdly, no pediatric patients were included in this study, which is a targeted population for radiation dose reduction strategies. The peculiarities of acquisition parameters for pediatric patients limited the type of patients included.

## Conclusion

The image quality of ULDCT-AIIR was not equivalent to that of SDCT-HIR in some anatomical parts; that is, the CT_mean_ and SNR were affected, while CNR and TBR were unaffected. The AIIR algorithm maintained the semiquantitative parameter values and diagnostic performance of the PET images. Overall, the image quality of ULDCT-AIIR was not superior to SDCT-HIR, but the AIIR algorithm decreased the image noise and relatively increased the SNR, which can be implemented under special circumstances in PET/CT examination.

## Supplementary Information

**Additional file 1:** The correlation analysis of PET parameters between the PET-HIR and PET-AIIR.

## Data Availability

The data that support the findings of this study are available from the corresponding author upon reasonable request.

## Abbreviations

PET: Positron emission tomography
CT: Computed tomography
ULDCT: Ultra-low-dose CT
SDCT: Standard-dose CT
HIR: Hybrid iterative reconstruction
AIIR: Artificial intelligence iterative reconstruction
SUV: Standardized uptake value
SNR: Signal-to-noise ratio
CNR: Contrast-to-noise ratio
CSF: Cerebrospinal fluid
ROI: Regions-of-interest
FDG: Fluorodeoxyglucose
ACCT: Attenuation–correction CT
OSEM: Ordered subset expectation maximization
TOF: Time of flight
PSF: Point spread function
TBR: Tumor-to-background ratio
