# Supplementary figures and images for: Ultra-low-dose CT reconstructed with the artificial intelligence iterative reconstruction algorithm (AIIR) in 18F-FDG total-body PET/CT examination: a preliminary study
Source: EJNMMI Phys. 2023 Jan 2;10:1. doi: 10.1186/s40658-022-00521-8 (PMC9807709; doi:10.1186/s40658-022-00521-8)

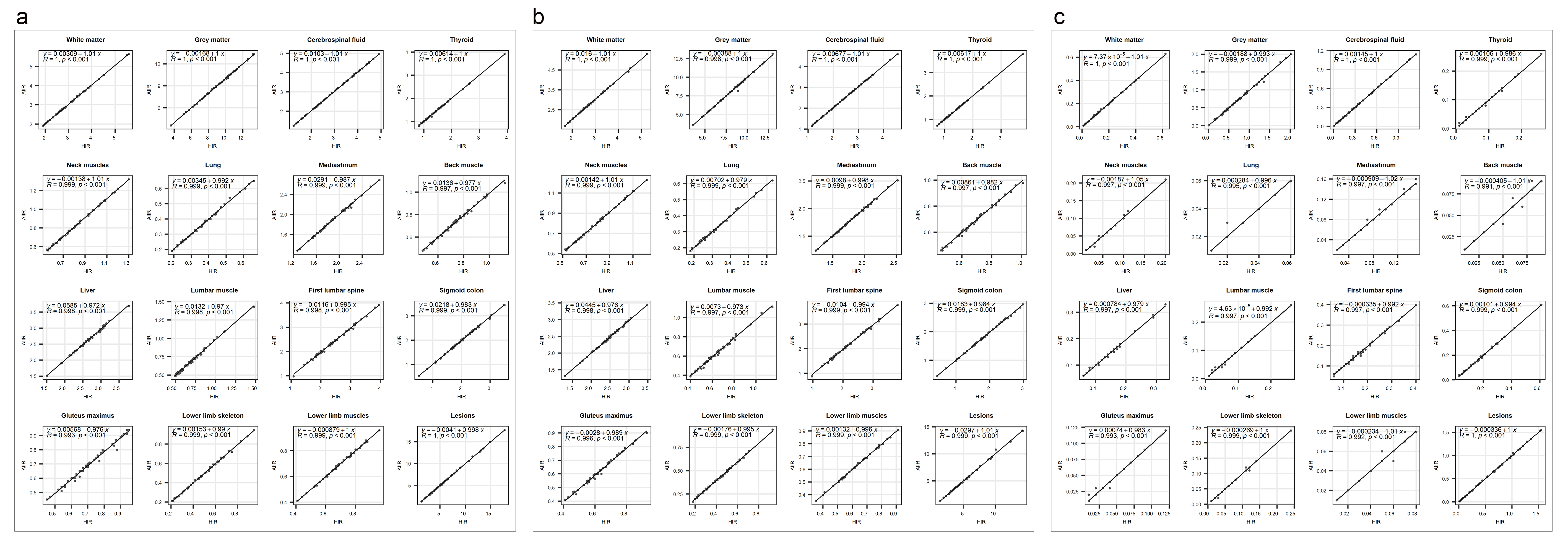

Supplement: Supplementary file 1 — Additional file 1: The correlation analysis of PET parameters between the PET-HIR and PET-AIIR. [file 40658_2022_521_MOESM1_ESM.jpg]
